# Energy-Saving Behaviours in Workplaces: Application of an Extended Model of the Theory of Planned Behaviour

**DOI:** 10.5964/ejop.v16i3.1893

**Published:** 2020-08-31

**Authors:** Luigina Canova, Anna Maria Manganelli

**Affiliations:** a Department of Philosophy, Sociology, Education and Applied Psychology, University of Padua, Padua, Italy; ZPID – Leibniz Institute for Psychology Information, Trier, Germany

**Keywords:** theory of planned behaviour, energy-saving behaviours in workplaces, cognitive attitude, affective attitude, habit

## Abstract

Individual energy-saving behaviours are crucial for reducing energy consumption, and research on the determinants of these behaviours has been increasing over the last decade. The aim of this study is to explore the determinants of two specific behaviours: ‘switching off non-essential lights’ and ‘completely switching off electronic devices’. An extended model of the theory of planned behaviour (TPB) has been used as the theoretical research framework. The extension was implemented by considering two components (affective and cognitive) of the attitude towards these behaviours and then adding habit as a new variable. A two-waves study was conducted in which a convenience sample of Italian workers completed a questionnaire measuring the TPB constructs in relation to the two energy-saving behaviours (Time 1). The participants then completed another questionnaire a month later to assess self-reports of these behaviours (Time 2). The inclusion of habit improved the predictive power of the TPB, and the extended model was found to explain 65.5% and 76.1% of the variance in intentions and 16.2% and 22.9% of the variance in behaviours. Cognitive attitude, subjective norm, perceived behavioural control, and habit were significantly related to intentions, and perceived behavioural control was the strongest predictor. Habit moderated some relationships between the TPB constructs and intentions. Behaviours were associated directly only with intentions. The results of this study support the efficacy of the TPB model in predicting target behaviours; they also suggest some strategies that can be followed to promote these energy-saving behaviours.

The proliferation of energy-consuming technologies (e.g., climate-control systems, personal computers) has resulted in an escalation in the use of energy, which has had a negative effect on the environment. This is reflected in the increased interest in environmental protection that can be achieved not only by using technologies that can improve energy efficiency but also by stopping the excessive consumption of natural resources and by practicing voluntarily individual behaviours that preserve the environment for future generations. To save energy, structural changes such as retrofitting buildings with energy-efficient characteristics are important starting points; however, they may not be enough to reduce energy consumption due to the ‘rebound effect’, i.e., they can even actually induce an increase in energy consumption. Therefore, motivating individuals to save energy may make an important contribution to reducing energy use.

Individual energy-saving behaviour has become an important research topic, but most studies have focused primarily on household energy-saving measures, and, to the best of our knowledge, little research has addressed the issue of individual energy-saving behaviour in workplaces (see the meta-analysis by Yuriev, Dahmen, Paillé, Boiral, & Guillaumie, 2020). The differences between domestic and organisational settings yield results at the residential level that are not immediately applicable to workplaces. In fact, these settings can make it easier or harder to perform pro-environmental behaviours (Maki & Rothman, 2017). For example, in a household setting, individual control is likely to be much higher than in an organisational setting such as an office, where behaviours may be shaped by the physical context (e.g., access to equipment control), by the social context (e.g., expectations and norms of other people), and by the organisational context (e.g., organisational culture and structure). Moreover, in the case of energy-saving behaviours, the costs are experienced more directly in the private sphere, while employees benefit only indirectly from the financial advantages of energy saving in workplaces (Littleford, Ryley, & Firth, 2014; Lülfs & Hahn, 2014). For example, a recent study by Littleford et al. (2014) found that people perform some, but not all, energy-saving behaviours consistently at home and work, but the study did not obtain any evidence to support the 'spillover' effect across workplace and household settings. Thus, to improve the efficacy of technical measures and organisational energy-saving policies, it is necessary to better understand employees’ opinions and behaviours. In fact, the impact of one individual’s energy-use decisions might seem small, but in the aggregate this impact will be significant. The present study aimed to explore the socio-psychological factors influencing two specific energy-saving behaviours in the workplace. The reference theoretical model was the theory of planned behaviour (TPB; Ajzen, 1991). The TPB model was extended by considering an additional variable, habit, which is not included in the original theory.

The TPB is a social-psychological model of behavioural decision-making (Ajzen, 1991). Briefly, this model predicts that the stronger the intention to engage in a behaviour, the more likely the behaviour will be performed. Three constructs determine behavioural intention: attitude towards the behaviour (i.e., the favourability a specific action has for an individual); subjective norm (i.e., the perception of expectations of relevant people that a specific action should be performed); and the perception of how easy or difficult it would be to perform the behaviour in a particular context (perceived behavioural control [PBC]). PBC is a construct that captures to what degree individuals believe they have control over their behaviours, including ability, time, skills, and opportunities to perform the behaviours. It is held to predict behaviour directly, along with intention, especially when the behaviour is not under complete volitional control. The TPB has been applied successfully in a wide range of fields, including pro-environmental behaviours (Klöckner, 2013).

The original TPB model has been extended in two main ways: a) the constructs (i.e., attitude towards the behaviour) have been decomposed in two or more dimensions and b) several factors have been explored as additional predictors or moderators of relationships in the TPB. Regarding attitude, two components have been distinguished: cognitive or instrumental and affective or experiential. The cognitive component reflects the act’s perceived instrumentality (i.e., its anticipated positive or negative consequences, perceived benefits and costs of the behaviour), whereas the affective component reflects the positive or negative experiences with the behaviour, as well as any emotion-based judgements about the behaviour (Fishbein & Ajzen, 2010). There is strong evidence that affective attitudes are more powerful predictors of a variety of health behaviours, especially those related to food choice (Blanchard et al., 2009; Canova, Bobbio, & Manganelli, 2020). As far as we know, the two components of attitude have received very little attention in research regarding environmental behaviours (e.g., Rhodes et al., 2015).

Regarding the second area of TPB expansion, i.e., integrating additional psychological factors and variables into the model to improve its predictive power, the most used variables have been past behaviour, personal or moral norms, self-identity, habit, and anticipated emotions.

The TPB has been used to examine pro-environmental behaviours and energy saving in household contexts, and it has been shown to be more successful in predicting behaviours, compared to other variables such as demographic (Abrahamse & Steg, 2009). In his meta-analysis, Klöckner (2013) noted that roughly 40% of all papers published in the environmental-psychological field had used the TPB as their basic theoretical framework, and analysing 56 different data sets with a variety of environmental behaviours showed that intentions, PBC, and habits were identified as direct predictors of behaviour. The strongest predictor was intentions followed by habit, while PBC had a weaker effect on behaviour. Attitudes, PBC, and personal and social norms (in descending order of their impact size) predicted intention.

In recent years, there has been an increasing interest in pro-environmental behaviours in workplaces, and some studies have applied the original or extended TPB models to predict energy-saving behaviours in this setting. Some research have considered, as target behaviours, specific pro-environmental behaviours, such as ‘switching off computer whenever leaving desk’, ‘using video-conferencing for any meeting’, ‘recycling as much waste produced at work as possible’ (Greaves, Zibarras, & Stride, 2013), and ‘printing smaller’, ‘not printing e-mails’, ‘switching off lights’, and ‘switching off monitors’ (Lo, Peters, van Breukelen, & Kok, 2014). In other research, composite indexes derived from several different pro-environmental behaviours were used (Blok, Wesselink, Studynka, & Kemp, 2015; Dixon, Deline, McComas, Chambliss, & Hoffman, 2015; Wesselink, Blok, & Ringersma, 2017), while other studies used a generic target behaviour: ‘energy saving in my company’ (Gao, Wang, Li, & Li, 2017; Zhang, Wang, & Zhou, 2014). In all the studies mentioned above, the theoretical model was the TPB, extended with additional predictors: personal or moral norms and descriptive norms (Gao et al., 2017; Lo et al., 2014); habit (Lo et al., 2014); organisational electricity-saving climate (Zhang et al., 2014); and perceived leadership support and exemplary behaviours (Blok et al., 2015; Wesselink et al., 2017). Only Greaves et al. (2013) explored further the specific antecedent beliefs of attitude (behavioural beliefs), subjective norms (normative beliefs), and PBC (control beliefs), with their results showing that most antecedent beliefs were significant in influencing behavioural intentions mediated via the TPB constructs. The quotas of explained variance in intentions ranged between 74% for the behaviour of ‘switching off monitors’ (Lo et al., 2014) and 22% for a composite index of several pro-environmental behaviours (Wesselink et al., 2017). Generally, attitude was associated with intentions, but regarding subjective norms and PBC, the results were less consistent, and the associations with intentions could range from very weak to strong.

Some limitations of TPB-based research described above have been highlighted (Yuriev et al., 2020). Firstly, most studies focused only on intention (e.g., Gao et al., 2017; Greaves et al, 2013; Zhang et al., 2014); further, those that focused on behaviour considered past behaviour as a proxy for future behaviour (e.g., Lo et al., 2014). However, when intention and behaviour are measured contemporaneously, a consistency bias is likely to inflate the correspondence between them (Hausenblas, Downs, Giacobbi, Tuccitto, & Cook, 2008). Furthermore, while some studies explored specific behaviours (e.g., ‘switching off lights’ or ‘not printing e-mails’), others considered composite indexes derived from many different pro-environmental behaviours, and yet others focused on a generic target behaviour such as ‘energy saving’. This imprecision in the definition of behaviours studied is problematic as the TPB seems unsuitable for exploring non-specific actions (Ajzen, 1991; Yuriev et al. 2020). Finally, the measures of TPB constructs did not follow the guidelines indicated by Fishbein and Ajzen (2010). All these discrepancies making it very difficult to compare results.

In our study, we considered habit as an additional predictor in the TPB model. Habits are viewed as playing a fundamental role in generating behaviour, and the term ‘habit’ is used widely in ordinary parlance to refer to frequent and persistent behaviour. Recently, Gardner (2015) defined habit as "a process by which a stimulus automatically generates an impulse towards action, based on learned stimulus-response associations" (p. 280). This definition includes cue-dependence, automaticity, and conditioned stimulus-response associations that depict habitual behaviour which, in turn, may be defined as "any action, or sequence of actions, that is controlled by habit" (Gardner, 2015, p. 282). Similarly, Hagger (2019) defined habit "as a specific action or behavioural tendency that is enacted with little conscious awareness or reflection, in response to a specific set of associated conditions or contextual cues" (p. 119). Sometimes, habit has been equated with the frequency of past behaviour, but there are good reasons for not accepting such analogy (Klöckner & Matthies, 2004). Past behaviour is the pattern of behaviour shown prior to the actual behaviour. It includes habitual components as well as intentional behaviour; it also includes repeated actions in addition to actions shown only once or occasionally. In contrast, a habit is the result of association between cues and behavioural patterns; this association is learnt by repeating the same behaviour under the same circumstances over and over again. Most scholars in defining habit added references to automaticity and to reduced deliberate thinking.

Verplanken and Aarts (1999) argue that habit should be introduced in the TPB as a further predictor and/or moderator of the intention-behaviour relationship. Most studies that have examined additive and interactive effects of habit are related to the domains of nutrition and physical activity (e.g., Gardner, de Bruijn, & Lally, 2011; Menozzi, Sogari, & Mora, 2017). In many cases, the results showed that habit explained additional quotas of behaviour variance and it moderated the relationship between intention and behaviour, so that intention had a reduced impact on behaviour when habit was strong. However, some studies failed to find evidence for the effect of ‘habit × intention’ interaction, in relation to physical activity (e.g., Murtagh, Rowe, Elliott, McMinn, & Nelson, 2012; Rhodes, de Bruijn, & Matheson, 2010).

Few studies have examined the effect of habit on relationships between the other constructs of the TPB (attitude, subjective norm, and PBC) and intention. In the study of de Bruijn et al. (2007), a multigroup analysis revealed that the impact of affective and cognitive attitudes and of subjective norm on intention increased when habit was low, while the impact of PBC increased when habit was high. Finally, Menozzi et al. (2017), in a study about healthy-eating behaviour, found that attitude and subjective norm had stronger impact on intention when habit was low.

In the ecological field, Klöckner and Matthies (2004) found that habit increased the amount of explained variance in behaviour and also weakened the association between personal norms and behaviour. Interestingly, despite also being recognised as an important determinant of pro-environmental behaviour, habit so far had mostly been neglected in research on energy-saving behaviours. A recent study by Wang, Lin, and Li (2018) analysed a TPB model extended with non-cognitive (personal moral norm and habit) and emotional factors (positive anticipated emotions). The target behaviour was ‘to save electricity in my home’. The model explained 26% of the variance in intention to save electricity and 30% of the variance in electricity-saving behaviour. Personal moral norm, attitude, PBC, habit, and positive anticipated emotion were positively and significantly associated with residents' intention to save electricity; habit also was associated with electricity-saving behaviour.

To the best of our knowledge, habit has not received extensive attention in the literature on pro-environmental behaviour in workplaces (for notable exceptions, see Lo et al., 2014). In fact, Lo et al. (2014) introduced habit in their study on organisational contexts. The examined behaviours were: ‘printing smaller’ and ‘not printing e-mails’ (printing behaviours), ‘switching off lights’ and ‘switching off monitors’ (switching behaviours). The habit was measured with two items selected from Verplanken's index (Self-Report Habit Index; Verplanken & Orbell, 2003). The hypothesised model was a TPB model extended with habit and personal norm. The explained variance of intention ranged from 50% for ‘not printing e-mails’ to 69% for ‘printing smaller’. With respect to behaviour, the quotas of explained variance ranged from 53% for ‘switching off lights’ to 74% for ‘printing smaller’ and ‘switching off monitors’. The examination of path coefficients showed that habit was a significant predictor of intention for all behaviours and that intention was the strongest direct predictor of printing behaviours, while habit was the strongest direct predictor of switching behaviours. Attitude had the strongest effect on intention for all behaviours, while the effects of subjective norm, personal norm, and perceived control were less consistent across behaviours. A limitation of this study was represented by the use of self-reported behaviour with reference to the past month as a proxy for future behaviour.

## The Present Study

The present study tested a TPB extended model aimed at improving our understanding of the factors affecting energy-saving behaviours in workplaces. Following the suggestions of Steg and Vlek (2009), we selected two target behaviours with the potential to provide significant environmental benefits that would ask individuals to change their behaviours in some way. The selected behaviours were: a) ‘switch off non-essential lights in the workplace (e.g., when you and your colleagues are leaving or when natural light is sufficient)’ and b) ‘completely switch off electronic devices (e.g., computer, computer screen, photocopier, and printer) without leaving them on stand-by at the end of a working day’. These behaviours also were the target behaviours in other studies: Lo et al. (2014) used ‘switch off non-essential lights in the workplace’ as a target behaviour in their study and Greaves et al. (2013) used ‘completely switch off electronic devices’.

Our hypothesised TPB model considered two attitude components: cognitive (or instrumental) and affective (or experiential; Fishbein & Ajzen, 2010). Some authors (Carrus, Passafaro, & Bonnes, 2008) emphasised that generally, the attitude-intention and attitude-behaviour relationships are complex, especially in the ecological domain. To address this problem, more attention should be paid to the different roles of affective and cognitive or instrumental attitudes in eliciting behaviour. Rhodes et al. (2015) showed that the effect of instrumental attitude on intention to recycle was greater compared with the impact of affective attitude. These authors argued that the instrumental attitude construct held some similarity to moral norms, which are used quite often in models of pro-environmental behaviours. Thus, considering the two components of attitude in extended TPB models would represent an interesting test of the importance of this distinction because pro-environmental behaviours may be motivated more by cognitive than affective evaluations compared with health behaviour. Our first hypothesis was the following:

H1. Cognitive attitude towards the behaviours would have stronger associations with intentions than affective attitude.

On the basis of the TPB, we proposed the subsequent hypotheses:

H2. Subjective norm will be positively associated with individuals' intentions to execute the two behaviours in workplaces during the next month.

H3. PBC will be positively associated to intentions and to future behaviours measured one month later.

H4. Intentions would predict self-reported future behaviours.

Furthermore, the model was augmented with an additional predictor: habit. The two behaviours considered in this study are likely under habitual control, at least to some extent, because they are characterised by both repetition (i.e., they are typically made every day even more than once) and situational stability (i.e., they can take place at the same time of day and in the same places). Thus, the aim of the study was to reveal whether the habit predicted, over and above the TPB constructs, behavioural intentions and behaviours and whether it acted as a moderator of the TPB relationships. Therefore, we offered the following hypotheses:

H5. Habit will significantly increase the proportion of explained variance in both intentions and behaviours and will be positively associated with intentions and behaviours.

H6. Habit would moderate the relationships between intentions and behaviours: the relationships are expected to be stronger when the habit is low.

Given the rare and mixed evidence for moderating the effects of habit on the relationships between habit itself and other TPB constructs, we did not specify any related hypotheses for these relations.

The present study has several contributions to make to the current literature. Firstly, it enriches and expands the research on energy-saving behaviours in workplaces considering an additional variable (habit), which is not included in the TPB theory and used very rarely in research on these specific behaviours. Further, this is the first study in the environmental field that investigated the moderating effects of habit with regard to all the relationships foreseen by the TPB. Secondly, although Fishbein and Ajzen (2010) acknowledge that the attitude construct is comprised of two components that represent affective and cognitive (or instrumental) evaluations of a behaviour, to our knowledge, these two components of attitude has not been considered in the case of energy-saving behaviours. Finally, as far as we know, this is the first study that has been conducted in the Italian context, which can contribute to the increase in geographical dispersion of research on pro-environmental behaviours in workplaces.

## Method

### Procedure and Participants

Two waves of data collection were organised. At Time 1, participants completed a structured questionnaire, including constructs measures of the hypothesised TPB model and socio-demographic variables. One month later, at Time 2, the same participants completed a second questionnaire, which included self-reported behaviour measures.

One hundred university students from two introductory courses in social psychology at Padua University collected data as part of a research experience assignment. Each student was asked to recruit four participants (adult workers at different organisations, companies, and industries) with whom they were acquainted and who lived in the same community or neighbourhood. The students distributed the instructions and the questionnaires for completion. They received no financial or other type of compensation. Participants took part on a voluntary basis and were informed that they could refuse to answer any questions and were also assured that all their responses would remain confidential. They completed the first questionnaire autonomously and returned it immediately. One month later, during scheduled appointments, they completed the second questionnaire and received a quick debriefing concerning the study’s aim. The completed questionnaires were carefully reviewed, and all questionnaires with missing values for the main variables were excluded. Of the 400 potential participants asked to complete the first questionnaire, usable data were received from 337 individuals, for a response rate of 84.3%. Of these, 295 participants (87.5%) also completed the second questionnaire.

The final sample comprised 295 participants: 166 were females (56.3%), 126 were males (42.7%), and 3 were participants who did not indicate their gender (1%). The mean age was 40.27 years, *SD* = 12.40, and range 20–64. Of the final respondents, 242 (82%) were office workers or technicians and 53 (18%) were self-employed workers, teachers, or blue-collar workers. As for job sectors, 206 (69.8%) were employed in the private sector and 86 (29.2%) in the public sector, with three (1%) not indicating their occupational sectors. Concerning education, 5.8% (*n* = 17) had only completed compulsory schooling, 61% (*n* = 180) had high-school-level education, and 32.5% (*n* = 96) had university degrees. Two (0.7%) participants did not indicate their education level. Most live in northeastern Italy (90.2%).

### Measures

At Time 1, participants completed a structured anonymous questionnaire including the measures of TPB constructs, habit, and socio-demographic variables. At Time 2, one month later, participants’ self-reported behavior measures were collected. The TPB constructs were assessed based on Fishbein and Ajzen’s (2010) guidelines, adapting items previously used in the Italian context (Canova & Manganelli, 2016; Canova et al., 2020). For both behaviours, the time reference was ‘the next month’. In the questionnaire, the two behaviours were presented in two different orders (see Supplementary Materials for items used to measure TPB constructs and habit).

### Data Analysis

Statistical analyses were performed using LISREL (Version 8.8) and SPSS (Version 22). LISREL was used to perform Confirmatory Factor Analysis to check the measurement model and whether all measures detected distinct constructs. The analyses were performed using the maximum likelihood method applied to covariance matrices for both behaviours separately. Goodness-of-fit was evaluated by means of the conventional indices that can be summarized as follows: χ^2^, χ^2^/*df*, CFI, RMSEA, and SRMR. Usually, a satisfactory model is denoted by χ^2^ not being significant, χ^2^/*df* ≤ 3, CFI ≥ .95, RMSEA ≤ .06, and SRMR ≤ .08 (Hu & Bentler, 1999). SPSS was used for descriptive analyses, Pearson’s correlation coefficients, reliability estimates through Cronbach’s alpha, and testing the hypothesised TPB extended models by moderated hierarchical regression analyses with bias-corrected bootstrap 95% confidence intervals (with 1000 resampling; see Supplementary Materials for research data and code).

## Results

### Measurement Model

The measurement models used included seven latent factors (cognitive and affective attitudes, subjective norm, PBC, habit, intention, and behaviour) and twenty-six indicators. The goodness-of-fit indices were satisfactory: ‘switching off lights’, χ^2^(278) = 723.04, *p* < .001, RMSEA = .07, 95% CI [.06, .08], CFI = .96, SRMR = .06; ‘switching electronic devices’, χ^2^(278) = 749.99, *p* < .001, RMSEA = .08, 95% CI [.07, .08], CFI = .97, SRMR = .06. All standardised factor loadings presented significant values (‘switching off lights’: from .55 to .95; ‘switching off devices’: from .55 to .94), showing that each latent factor was defined by its own indicators. The correlations among the latent factors were all significant (except for the correlation between affective attitude and behaviour in the case of ‘switching off devices’, where ϕ = .09); for both behaviours, the most correlated constructs were intention and PBC (‘switching off lights’: ϕ = .82; ‘switching off devices’: ϕ =. 87). In every case, the 95% confidence intervals, obtained by considering two standard errors above and below the coefficients, did not include the perfect correlation (i.e., 1.00), thus supporting the fact that all measures captured distinct constructs (Bagozzi, 1994).

### Descriptive Statistics

Cronbach’s alpha, means and standard deviations of TPB constructs, and habit and self-reported behaviours have been shown in Table 1. Cronbach’s coefficients were satisfactory for all measures, and composite scores for each construct were computed. Respondents intended to carry out the two energy-saving behaviours over the next month. The evaluation of the acts (cognitive attitude) was positive and affective reactions (affective attitude) were favourable. Participants stated that they perceived good control (PBC) over the two energy-saving behaviours and that they felt quite strong social pressure from significant important others. They also declared a good level of habit for both behaviours. Finally, at Time 2, behaviours were carried out quite often during the last month.

**Table 1:** Reliability Coefficients, Descriptive Statistics, and Correlations Between the TPB Constructs (N = 295)

Construct	Switching off lights			Switching off electronic devices			1	2	3	4	5	6	7
	α	*M*	*SD*	α	*M*	*SD*							
1. Cognitive attitude^a^	.79	6.52	0.69	.79	6.41	0.84	-	.51	.35	.35	.52	.42	.25
2. Affective attitude^a^	.78	5.64	1.05	.78	5.60	1.03	.46	-	.30	.25	.35	.31	.10^c^
3. Subjective norm^a^	.83	5.68	1.36	.87	5.56	1.46	.22	.18	-	.46	.51	.49	.29
4. PBC^a^	.82	5.77	1.23	.87	5.72	1.43	.48	.31	.25	-	.74	.57	.36
5. Intention^a^	.87	6.02	1.00	.91	5.92	1.24	.58	.41	.31	.68	-	.74	.45
6. Habit^a^	.96	5.70	1.43	.96	5.62	1.59	.36	.32	.26	.51	.61	-	.34
7. Behaviour^b^	.90	3.81	0.75	.92	3.67	0.93	.21	.14	.18	.24	.36	.31	-

*Note.* Lower diagonal: Switching off lights; Upper diagonal: Switching off electronic devices. PBC = Perceived behavioural control.

^a^Measured on a 7-point scale. ^b^Measured on a 5-point scale. ^c^Not significant, other correlations are significant *p* < .01.

Zero-order correlations between all variables included in the regression analyses are reported in Table 1. For both behaviours, the highest correlations (large effect sizes) were those between PBC and intention, and between habit and intention. Moreover, the correlations between cognitive attitudes and intention showed similar effect sizes. The other correlations among the TPB constructs are regarded as medium or small effect sizes.

### Behavioural Intentions

To test our hypotheses, we conducted two moderated hierarchical regression analyses, with intention as the dependent variable. The TPB constructs (cognitive and affective attitude, subjective norm, and PBC) were entered in the first step, and habit was entered in the second step. In the third step, the two-way interaction terms between habit and TPB variables were entered. All the variables were standardized (see Supplementary Materials for Tables and Figures showing the results of regression analyses).

Regarding the behaviour of ‘switching off non-essential lights’, in Step 1, cognitive attitude (*b* = 0.28, *p* < .001), affective attitude (*b* = 0.11, *p* < .05), subjective norm (*b* = 0.11, *p* < .05), and PBC (*b* = 0.49, *p* < .001) accounted for 56.4% of the variance in intention and were associated significantly with this outcome variable, with PBC emerging as the strongest predictor. In Step 2, the inclusion of habit contributed a further 5.7% to the variance explained in intention. Cognitive attitude (*b* = 0.25, *p* < .001), subjective norm (*b* = 0.08, *p* < .05), PBC (*b* = 0.37, *p* < .001), and habit (*b* = 0.29, *p* < .001) were significant predictors in this step. In the last step, the interaction terms between habit and the TPB constructs were introduced, accounting for significant incremental variance in intention (3.4%). The overall model explained 65.5% of the variance in intention. In this model, cognitive attitude (*b* = 0.28, *p* < .001), subjective norm (*b* = 0.10, *p* < .01), PBC (*b* = 0.31, *p* < .001), habit (*b* = 0.20, *p* < .001), and one interaction term, given by PBC × habit, were significantly associated with intention (*b* = -0.16, *p* < .001); PBC and cognitive attitude were the strongest predictors. Significant interaction was examined more closely by calculating the regression slopes from the full equation at the high and low (+1 *SD* and -1 *SD*) levels of habit (Dawson, 2014). The relationship between PBC and intention was stronger for individuals with low habit (*b* = 0.47, *t* (285) = 8.64, *p* < .0001) than for those with high habit (*b* = 0.15, *t* (285) = 2.76, *p* < .007).

Regarding the behaviour of ‘switching off electronic devices’, the core variables of the TPB explained 64.6% of the intention variance. Cognitive attitude (*b* = 0.25, *p* < .001), subjective norm (*b* = 0.15, *p* < .01), and PBC (*b* = 0.58, *p* < .001) were associated significantly with intention. PBC was the strongest predictor. Habit, introduced in Step 2, contributed a further 8.4% to the explained intention variance and was associated significantly with intention (*b* = 0.38, *p* < .001). The interaction terms in Step 3 made a significant contribution (3.1%) to explaining the variance, and the overall model explained 76.1% of the intention variance. Cognitive attitude (*b* = 0.09, *p* < .05), PBC (*b* = 0.42, *p* < .001), habit (*b* = 0.39, *p* < .001), and two interaction terms − cognitive attitude × habit (*b* = -0.11, *p* < .05) and subjective norm × habit (*b* = 0.14, *p* < .05) − were associated significantly with intention; PBC and habit were the strongest predictors. The relationship between cognitive attitude and intention was significant for individuals with low habit (-1 *SD*; *b* = 0.20, *t* (285) = 3.62, *p* < .0001) and it was not significant for those with high habit (+1 *SD*; *b* = -0.01, *t* (285) = -.26, *ns*). The relationship between subjective norm and intention was significant for individuals with high habit (*b* = 0.19, *t* (285) = 3.45, *p* < .002) and it was not significant for those with low habit (*b* = -0.09, *t* (285) = -1.63, *ns*).

### Self-Reported Behaviours

In Steps 1 and 2 of hierarchical regression analyses, direct effects of the TPB core constructs and habit were considered. Intention was introduced in Step 3. Finally, interaction terms among the TPB constructs, intention, and habit were introduced in the equations in Step 4.

Regarding the behaviour ‘switching off lights’, only habit had a significant direct effect (*b* = 0.22, *p* < .05) on self-reported behaviour (Step 2), which did not appear to be significant when controlling for intention (*b* = 0.29, *p* < .01; Step 3). Thus, intention mediated the relationship between habit and self-reported behaviour. The indirect effect is significant (*b* = 0.08, *z* = 2.96, *p* < .004; 95% CI [.02, .17]). The interaction terms did not contribute to incremental variance in behaviour. The TPB constructs and habit explained 15.1% of the variance in behaviour.

Regarding the behaviour ‘switching off electronic devices’, direct effects of PBC emerged at Step 1 (*b* = 0.26, *p* < .01) and at Step 2 (*b* = 0.20, *p* < .05), which did not appear to be significant when controlling for intention (*b* = 0.38, *p* < .01; Step 3). Thus, intention mediated the relationship between PBC and self-reported behaviour. The indirect effect was significant (*b* = 0.17, *z* = 3.59, *p* < .0004; 95% CI [.06, .28]). The introduction of interaction terms did not increase the quota of explained variance in behaviour significantly, and none of the interaction terms was significant. In this case, the TPB constructs and habit explained 21.5% of the variance in behaviour.

## Discussion

The findings of this study sustained the validity of the TPB in predicting energy-saving behaviours in workplaces, providing further evidence of the efficacy of this model. The results showed that intention was the only direct predictor of the two behaviours. In turn, intention was associated with cognitive attitude, subjective norm and PBC. Hence, the three hypotheses H1, H2, and H4 were supported. Intention performs as an integrative variable gathering the impact of cognitive attitude, subjective norm, and PBC. Hypothesis H3 was partially supported. In fact, PBC was the strongest predictor of intention to switch off both lights and electronic devices, but it is was not directly associated with behaviours. In the literature, the overall evidence concerning the efficacy of PBC in directly predicting behaviour is not consistent. Additionally, it has been suggested that PBC represents not only perceived physical control but also perceived difficulty or even social appropriateness, which might clarify why PBC is an important construct to consider when predicting people's intentions to conserve energy in public settings. Some studies suggest that perceived control is more important in office settings (e.g., Littleford et al., 2014) or in other contexts outside of the home (e.g., Maki & Rothman, 2017) and is perhaps less relevant in one's own home. However, the finding that intention predicted behaviour but PBC did not suggests that, for the energy-saving behaviours considered in this study, while PBC serves to shape intention—even after controlling for the effects of habit—it does not predict behaviours that seem to be under individual volitional control.

The core variables of TPB explained high quotas of variance intention (56.4% and 64.6%). These percentages of accounted variance, similar to those reported by Greaves et al. (2013) and Lo et al. (2014), might be because in our research, we focused on highly specific behaviours that may not be open to alternative interpretations. Therefore, the possibility of incompatibility between the person's representation of the attitude target and the actual target, towards which the attitude and intention are directed, was very low. Affective and cognitive attitudes towards the behaviours were favourable, but only the cognitive component of attitude was associated with intention. Cognitive attitude reflects the anticipated positive or negative consequences and perceived benefits and costs of the behaviour. It is interesting to note the differences in effects that the two components of attitude have, compared with the behaviours of food choice and consumption. For these behaviours, affective attitude, i.e., the pleasantness of foods, emerged usually as a predictor of intention to consume a certain type of food, as consumption of most liked food usually leads to enjoyment (Canova et al., 2020). The two components have not been studied specifically in the energy-saving domain, but the cognitive component has considerable similarity with moral norm, given the environmental impact of energy-saving behaviours. Moreover, our results, in line with those of Rhodes et al. (2015), support the position that the two components of attitude have different relationships with intention, even in the context of pro-environmental behaviours.

Adding habit increased the portion of variance explained in intentions and behaviours. In the case of behaviours, the increase, although significant, was lower. The quotas of additional variance explained in intentions were similar to those reported in the literature. However, the relatively low explained variance of behaviours, when habit was also considered, indicated that other motivational, volitional, and/or environmental factors need to be considered for predicting energy-saving behaviours. The intention-behaviour gap, that is, the difference between a participant’s intention to act in a certain way and the actual behaviour, is very common in TPB-based studies (Yuriev et al., 2020). Some reasons for this gap have been highlighted: a) events that occurred between assessment of intentions and assessment of behaviours may have produced changes in intention, b) unanticipated obstacles may have prevented the individuals from carrying out their intentions, and c) measures of actual behaviour can be fallible both with respect to reliability and with respect to construct validity. Hence, validate measures of pro-environmental behaviours would allow researchers to identify and evaluate actual behaviours with more precision (de Leeuw, Valois, Ajzen, & Schmidt, 2015). Habit was a strong predictor of intention, but it was not directly associated with behaviours, and its effect was mediated by intention in the case of switching off lights. Thus, the hypothesis H5 was partially supported. Our results on the relationship between habit and behaviour are different from those of other studies (e.g., Lo et al., 2014); however, we must bear in mind that in our research, the behaviours were surveyed at a later stage than the one in which habit and intentions were detected. Finally, habit did not moderate the intention-behaviour relationship. Therefore our hypothesis H6 was not supported. Our findings—in line with Murtagh et al. (2012), do not support the behaviourist view that habit diminishes the effects of rational decision-making (e.g., intention) on behaviour—suggest that motivational and habitual processes have complementary but independent effects on the two switching behaviours. Nevertheless, habit intervened to modify the strength of some relationships between predictors and intentions: a) if individuals had low habit in switching off lights, the intention to do it was more likely if they perceived the behaviour under their control; b) still, if individuals had low habit of completely switching off their electronic devices, the intention to do it was more likely if they developed a strong cognitive attitude, i.e., if they positively evaluated the consequences of their behaviour; c) finally, if individuals had a poor habit of switching off electronic devices, social pressure had no effect on the formation of intention.

This study has a few limitations that should be considered in generalising its results and in choosing directions for future research and interventions. First, we interviewed a convenience sample of workers from different organisations; therefore, the results may not be generalisable. Moreover, we have used single-source self-reported data, which can overestimate energy-saving behaviours. Although this is a widespread approach, some scholars have criticised it. Indeed, self-reported data, especially for behaviours, are vulnerable to social desirability, social approval biases, and retrieval inaccuracy. Future research should include more objective measures, such as tracking of energy use, e.g., via smart plugs (Ruepert et al., 2016). Moreover, although our study considers a prospective measure of behaviour, it is correlational in design and does not allow the inference of causal relations.

In this study, organizational factors or context characteristics were not considered. Future studies could examine which contextual factors affected the likelihood that the TPB constructs or other variables predicted different types of pro-environmental behaviours in workplaces. For example, Lo et al. (2014) found that the number of office co-workers had a negative effect on the behaviour of switching off lights, over and above the TPB constructs. The number of co-workers also had negative effects on attitude and on perceived control over switching off lights. Future research should examine other contextual factors more deeply, e.g., the green-work climate, i.e., employees’ perceptions regarding organisational characteristics and behavioural norms within a company that pertain to environmental sustainability (Norton, Zacher, & Ashkanasy, 2014); perceived organisational support for the environment (Lamm, Tosti-Kharas, & King, 2015); and workplace leaders’ own habits/behaviours and support for green initiatives (Dumitru et al., 2016; Wesselink et al., 2017).

Finally, beliefs associated with attitude towards the behaviours (behavioural beliefs), subjective norms (normative beliefs), and PBC (control beliefs; Ajzen, 1991) were not included. Thus, future studies could examine these beliefs to fully understand behavioural decisions and develop interventions to promote energy-saving behaviours in workplaces.

Nevertheless, our results have certain implications for companies on how they can encourage their employees to adopt energy-saving practices in workplaces. Company management should recognise the role of employees in energy saving and improve their perception of organisational benefits (e.g., showing how much energy and money employees can save for the company if they engage in energy saving). PBC significantly promotes the intention to save energy. Creating a feeling of self-efficacy, i.e., the ability to perform the act, is important in developing energy saving intentions. Thus, interventions to increase PBC are very important. Individuals should be supported to overcome obstacles and barriers by creating favourable circumstances (e.g., providing an energy-saving manual, providing easy access to equipment controls for turning off lights and devices), which should be introduced to develop the intention to perform energy-saving behaviours.

Moreover, low habit to perform energy-saving behaviours in workplaces and low PBC can obstacle the formation of intention and, consequently, the performance of the behaviour. To reduce energy consumption in workplaces, energy-saving behaviours should become habitual in everyday life, which may require the deactivation old habits, particularly for frequent behaviours in relatively stable contexts (Lülfs & Hahn, 2014). In fact, successful habit modification interventions involve breaking up the contextual factors that automatically cue habit execution and introducing changes in the context.

### Conclusion

This study expands the literature on energy-saving behavioural intentions and self-reported behaviours. Overall, we found that the TPB could be useful in understanding energy-saving intentions and behaviours in workplaces. Intentions were the only direct predictor of behaviours. Furthermore, the results indicated that cognitive attitude (perceived instrumentality of the act), subjective norm, PBC, and habit were associated significantly with intentions, while affective attitude was not associated with them. This result is interesting and new in literature on energy conservation in workplaces, supporting the idea that the two components of attitude have different relationships with intentions in different behavioural contexts. Habit increased the portion of variance explained in intentions, but was not directly associated with behaviours and did not moderate the intention-behaviour relationships. The study has several practical implications; nevertheless, it suggests that companies and management, to improve these pro-environmental behaviours among their workforces, should remove obstacles and barriers to their execution.

## Data Availability

A dataset for this study is freely available (see the Supplementary Materials section).

## Notes

For this article the following supplementary materials are available:

- Research data: SPSS dataset (Canova & Manganelli, 2020a).
- Code: SPSS data analysis script (Canova & Manganelli, 2020b).
- Measures of research constructs, Tables 2 and 3, and Figures 1, 2 and 3 showing the results of regression analyses (Canova & Manganelli, 2020c).

## References

[sp1_r1] Canova L. Manganelli A. M. (2020a). Supplementary materials to "Energy-saving behaviours in workplaces: Application of an extended model of the theory of planned behaviour" [Research data]. PsychOpen. 10.23668/psycharchives.2798 PMC7909501 33680189

[sp1_r2] Canova L. Manganelli A. M. (2020b). Supplementary materials to "Energy-saving behaviours in workplaces: Application of an extended model of the theory of planned behaviour" [Code]. PsychOpen. 10.23668/psycharchives.2799 PMC7909501 33680189

[sp1_r3] Canova L. Manganelli A. M. (2020c). Supplementary materials to "Energy-saving behaviours in workplaces: Application of an extended model of the theory of planned behaviour" [Measures, figures, tables]. PsychOpen. 10.23668/psycharchives.3121 PMC7909501 33680189

